# ﻿*Sinocrassulaobliquifolia* (Crassulaceae), a new species from China

**DOI:** 10.3897/phytokeys.255.142079

**Published:** 2025-04-14

**Authors:** Rong-Juan Li, Jing Zhao, Shao-Li Fang, Chuan-Jie Huang, Miao Luo, Zhao-Rong He, Xin-Mao Zhou, Jia-Guan Wang

**Affiliations:** 1 School of Ecology and Environmental Science & School of Life Science, Yunnan University, Kunming, 650504, Yunnan, China Yunnan University Kunming China; 2 Kunming Coal Design and Research Institute, Baita Road, Panlong District, Kunming, 650011, Yunnan, China Kunming Coal Design and Research Institute Kunming China

**Keywords:** Hengduan mountains, phylogeny, rosette, species diversity, taxonomy

## Abstract

Based on a comprehensive morphological and molecular data analysis, we have confirmed and described a new species within the genus *Sinocrassula*, which is distributed in Sichuan Province, China. Morphologically, the new species resembles *Sinocrassuladiversifolia* and *S.indica*, but it is distinctly different from them in its asymmetrical leaves, leaf apex with glands, triangular petals that are white at the base and adorned with dense purple-red stripes and spots on the surfaces upward, and rectangular nectar scales. A phylogenetic analysis utilizing four plastid markers and one nuclear marker supports the conclusion that the new species is sister to *S.ganluoensis*.

## ﻿Introduction

Crassulaceae are a morphologically diverse and systematically complex group of angiosperms, comprising 35 genera and approximately 1,400 species (Berger 1930; Eggli 2003; Thiede and Eggli 2007; Messerschmid et al. 2020). Known for their heat resistance and rapid growth, members of the Crassulaceae family are highly adaptable and easy to cultivate. This adaptability makes them excellent for soil improvement and essential components in vertical gardens and green roofs (Zhu 2015). *Sinocrassula* A. Berger is a small genus within the family Crassulaceae, comprising approximately ten species distributed across various regions, including Bhutan, China, India, Nepal, Pakistan, Sikkim, and Vietnam (Wang et al. 2012, 2022; Fu and Ohba 2013; Averyanov et al. 2014). Despite the distinct characteristics of *Sinocrassula*, defining monophyletic groups within the genus has proven to be extremely challenging due to the frequent intergradation of morphological traits among taxa, as well as the extensive diversity in morphology and growth habits (Fu and Ohba 2013). Some species of *Sinocrassula* (e.g., *S.indica*) have been used as traditional medicine, or enjoyed as a vegetable and infused in herbal teas (Zhao et al. 2004; Xie and Yoshikawa 2012). In recent years, an increasing number of species of *Sinocrassula* have been discovered in Asia (e.g., *S.jiaozishanensis* Chao Chen, J. Guan Wang & Z.R. He, *S.vietnamensis* Aver. & V.V. Byalt) (Averyanov et al. 2014; Wang et al. 2022). The discovery of new species may provide more medicinal resources and increase the species diversity of *Sinocrassula*.

In July 2023, we conducted two field surveys in Sichuan Province, where we identified two populations of *Sinocrassula* in Shimian and Hanyuan counties. After carefully culturing the plants and performing a morphological study, we discovered that these populations are distinctly different from all other species within the genus. Additionally, we conducted a phylogenetic analysis, which indicated that these populations form a well-supported clade that is sister to *S.ganluoensis* (Li et al. 2024). We propose those populations as a new species of *Sinocrassula*.

## ﻿Materials and methods

### ﻿Morphological studies

The living plants of the new species were cultivated in the greenhouse at Yunnan University. Plant morphologies were photographed using a Nikon SMZ1270 stereo microscope (Nikon). Morphological comparisons among the new species and its related species were from field observations, herbarium investigations, and the literature (e.g., Fu and Ohba 2013; Averyanov et al. 2014; Wang et al. 2022). Voucher specimens of the new species have been deposited in the Herbarium of Yunnan University (YUKU; herbarium acronyms follow Index Herbarium by Thiers 2024).

### ﻿Taxonomic sampling

In order to clarify the phylogenetic position of the new species, two samples of the new species and its putative closely related taxa of *Sinocrassula* from China were included in the phylogenetic analysis. In total, 22 accessions representing 12 species of *Sinocrassula* were used for the phylogenetic analysis. Based on previous phylogenetic studies (Messerschmid et al. 2020; Wang et al. 2022), *Kungiaaliciae* (Raym.–Hamet) K. T. Fu was selected as the outgroup.

### ﻿DNA extraction, amplification, and sequencing

Total genomic DNA was extracted from silica-dried material using the TIANGEN plant genomic DNA extraction kit (TIANGEN Biotech., Beijing, China) following the manufacturers’ protocols. Four chloroplast DNA markers (*psb*A-*trn*H, *trn*L-F, *rbc*L, *mat*K)] and one nuclear marker (ITS) were amplified and sequenced using previous primers and protocols (Wang et al. 2022).

### ﻿Phylogenetic analyses

Sequences from GenBank and the newly generated data (Table 1) were aligned using MAFFT v7.450 (Katoh and Standley 2013), followed by manual refinement in BioEdit (Hall 1999). Single alignments were concatenated to a matrix using the ‘Concatenate Sequence’ plugin in PhyloSuite v1.2.2 (Zhang et al. 2020). ModelFinder (Kalyaanamoorthy et al. 2017) was used to select the best-fitting likelihood model for Maximum Likelihood (ML) and Bayesian Inference (BI) using the bias-corrected Akaike information criterion (AICc). Maximum likelihood bootstrapping was performed with 5,000 rapid bootstrap (BS) analyses followed by a search for the best-scoring tree in a single run through IQ-tree v2.1.2 (Nguyen et al. 2015). Bayesian inference was conducted for the combined dataset using MrBayes v3.1.2 (Huelsenbeck and Ronquist 2001) with two runs of four Markov chain Monte Carlo (MCMC) chains, each beginning with a random tree and sampling every 1000 generations for 20,000,000 generations. Finally, the concatenated trees were generated and visualized with their Maximum-Likelihood Bootstrap Support values (ML-BS) and Bayesian Inference Posterior Probability (BI-PP) in Figtree v1.4.3 (Rambaut 2017).

**Table 1.** Species information and corresponding GenBank accession numbers of *Sinocrassula* and close relative genus used in this study.

Species	Voucher	Location	*trn*L-*trn*F	*psb*A-*trn*H	*rbc*L	ITS	*mat*K	Reference
*Kungiaaliciae*	Mayuzumi CH00061 (TI)	Sichuan, China	AB480632	–	–	AB480591	–	Mayuzumi and Ohba 2009
*Sinocrassulaambigua*	Chen et al. YUS12973 (YUKU)	Deqin, Yunnan, China	PQ629032	PQ629054	PQ629039	PQ611189	PQ629047	Xu et al. 2025
*S.ambigua*	Chen et al. YUS12672 (YUKU)	Deqin, Yunnan, China	PQ629030	PQ629055	PQ629038	PQ611188	PQ629046	Xu et al. 2025
*S.ambigua*	Chen et al. YUS6698 (YUKU)	Deqin, Yunnan, China	PQ629035	PQ629059	PQ629040	PQ611190	PQ629048	Xu et al. 2025
*S.densirosulata*	Chang XC19075 (SZ)	China	MW206800	MW206800	MW206800	–	MW206800	Unknown
*S.diversifolia*	Chen et al. YUS9407 (YUKU)	Gongshan, Yunnan, China	**PQ629070**	**PQ629074**	**PQ629066**	**PQ623396**	**PQ629062**	This study
*S.diversifolia*	Chen et al. YUS9477 (YUKU)	Gongshan, Yunnan, China	**PQ629071**	**PQ629075**	**PQ629067**	**PQ623397**	**PQ629063**	This study
*S.ganluoensis*	Zhao et al. YUS6699 (YUKU)	Ganluo, Sichuan, China	PQ505691	PQ505693	PQ505695	PQ496498	PQ505697	Li et al. 2024
*S.ganluoensis*	Zhao et al. YUS13920 (YUKU)	Kangding, Sichuan, China	PQ505692	PQ505694	PQ505696	PQ496499	PQ505698	Li et al. 2024
*S.holotricha*	Zhao et al. YUS13475 (YUKU)	Jiulong, Sichuan, China	PQ629034	PQ629056	PQ629042	PQ611192	PQ629050	Xu et al. 2025
*S.holotricha*	Zhao et al. YUS12867 (YUKU)	Yuexi, Sichuan, China	PQ629031	PQ629057	PQ629043	PQ611193	PQ629051	Xu et al. 2025
*S.indica*	zjq20160061 (SANU)	Xizang, China	MN794334	MN794334	MN794334	–	MN794334	Zhao et al. 2020
S.indicavar.obtusifolia	Wang et al. YUS13936 (YUKU)	Deqin, Yunnan, China	PQ505699	PQ505701	PQ505703	PQ496500	PQ505705	Li et al. 2024
S.indicavar.obtusifolia	Wang et al. YUS13959 (YUKU)	Deqin, Yunnan, China	PQ505700	PQ505702	PQ505704	PQ496501	PQ505706	Li et al. 2024
*S.jiaozishanensis*	Chen et al. JZS001 (YUKU)	Luquan, Yunnan, China	MZ343264	MZ343262	MZ343263	–	MZ343261	Wang et al. 2022
*S.jiaozishanensis*	Chen et al. JZS002 (YUKU)	Luquan, Yunnan, China	MZ343269	MZ343267	MZ343268	–	MZ343266	Wang et al. 2022
*S.jiaozishanensis*	Chen et al. YUS5900 (YUKU)	Luquan, Yunnan, China	PQ629036	PQ629058	PQ629044	PQ611194	PQ629052	Xu et al. 2025
*S.obliquifolia*	Zhao et al. YUS9064 (YUKU)	Hanyuan, Sichuan, China	**PQ629072**	**PQ629076**	**PQ629068**	**PQ623398**	**PQ629064**	This study
*S.obliquifolia*	Huang et al. YUS9366 (YUKU)	Shimian, Sichuan, China	**PQ629073**	**PQ629077**	**PQ629069**	**PQ623399**	**PQ629065**	This study
*S.yunnanensis*	Chen s.n. (HIB)	Yunnan, China	–	–	–	KC988288	KC988295	Chen et al. 2014
*S.yunnanensis*	Mayuzumi C00115 (TI)	Yunnan, China	AB480669	–	–	AB088582	–	Mayuzumi and Ohba 2004
*S.yunnanensis*	Chen et al. YUS6697 (YUKU)	Heqing, Yunnan, China	PQ629037	PQ629060	PQ629045	PQ611195	PQ629053	Xu et al. 2025
*S.yunnanensis*	Chen et al. YUS13776 (YUKU)	Heqing, Yunnan, China	PQ629033	PQ629061	PQ629041	PQ611191	PQ629049	Xu et al. 2025

Note. Accession numbers in bold indicates newly generated data for this study.

## ﻿Results and discussion

**Figure 1.**
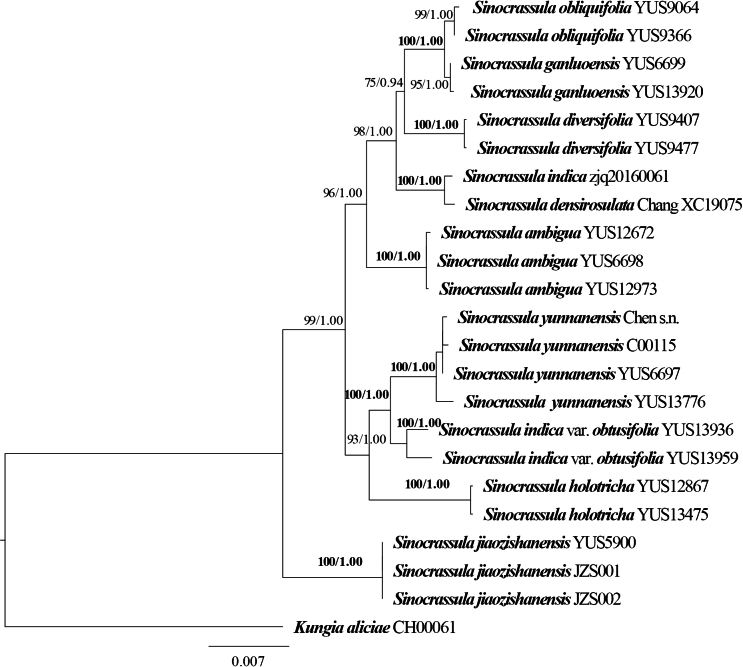
The maximum likelihood phylogeny of *Sinocrassula* and its allies based on four chloroplast markers (*psb*A-*trn*H, *trn*L-F, *rbc*L, and *mat*K) and one nuclear marker (ITS). Values associated with branches are Maximum Likelihood Bootstrap Support (ML-BS) and Bayesian Inference Posterior Probability (BI-PP). Bold indicated it received full ML-BS and BI-PP support.

A total of 23 accessions representing 12 species from the genus *Sinocrassula*, along with one outgroup (*Kungiaaliciae*), were included in the phylogenetic analysis. The concatenated dataset was 6,433 bp in length and GTR + F + I + G4 was selected as the best evolutionary model of nucleotide substitutions. The inferred phylogenetic trees from the ML and BI analyses revealed identical topologies. Two samples of the new species formed a highly supported clade (ML-BS = 99; BI-PP = 1.00, Fig. 1), and were found to be sister to *Sinocrassulaganluoensis* (ML-BS = 100, BI-PP = 1.00, Fig. 1). Then, they together are sister to *S.diversifolia* (ML-BS = 74, BI-PP = 0.94, Fig. 1). Although the new species is closely related to *S.ganluoensis* and *S.diversifolia*, it can be easily distinguished from these two species by its asymmetrical leaves and triangular petals, which are white at the base with dense purple-red stripes and spots on the both surfaces upward. Morphologically, the new species is most similar to *S.indica* (Table 2). However, the phylogenetic analysis showed that they have a distant relationship (Fig. 1).

**Table 2.** Morphological comparison of *Sinocrassuladiversifolia*, *S.ganluoensis*, *S.indica*, and *S.obliquifolia*.

Character	*S.diversifolia*	*S.ganluoensis*	*S.indica*	*S.obliquifolia*
Life cycle	—	Perennial	Biennial	Biennial
Plant surface	Glabrous, many parts purple-spotted	Glabrous	Glabrous	Glabrous, many parts purple-spotted
Basal leaves	Rosette lax, many parts brown-spotted, broadly obovate	Rosette compact, orbicular- lanceolate	Rosette, spatulate-oblong	Rosette, asymmetrical leaves ovoid to lanceolate
Stem leaves	Alternate, dimorphic	Alternate, Linear, lanceolate	Alternate, broadly oblanceolate, subobovate, ovate-orbicula	Alternate alternate, lanceolate, many parts with purple spots
Sepals	Triangular-lanceolate	Ovate-lanceolate	Broadly triangular	Broadly triangular
Bracts	Resembling distal stem leaves but smaller	Lanceolate	Resembling distal stem leaves but smaller	Lanceolate
Inflorescences	Corymbiform	Corymbiform	Paniculate, often corymbiform	Corymbiform
Length of flowering stems	—	5–11.5 cm	5–60 cm or shorter	10–18 cm
Petals	Lanceolate, yellow, spotted with purple	Broad triangular, yellowish, deeply purplish red upward	Lanceolate to ovate, red, reddish, yellow or greenish yellow	Triangular, base white, spotted with purple upward
Nectar scales	Broadly quadrate	Quadrate	Quadrate, apex emarginate	Rectangler
Nectar scales size	0.3–0.5 × 0.5–0.7 mm	0.5 × 0.9 mm	—	0.3–0.5 × 0.2–0.3 mm
Length of styles	0.5–1.0 mm	0.6–1 mm	Less than 1.0 mm	1.0–1.5 mm

**Figure 2.**
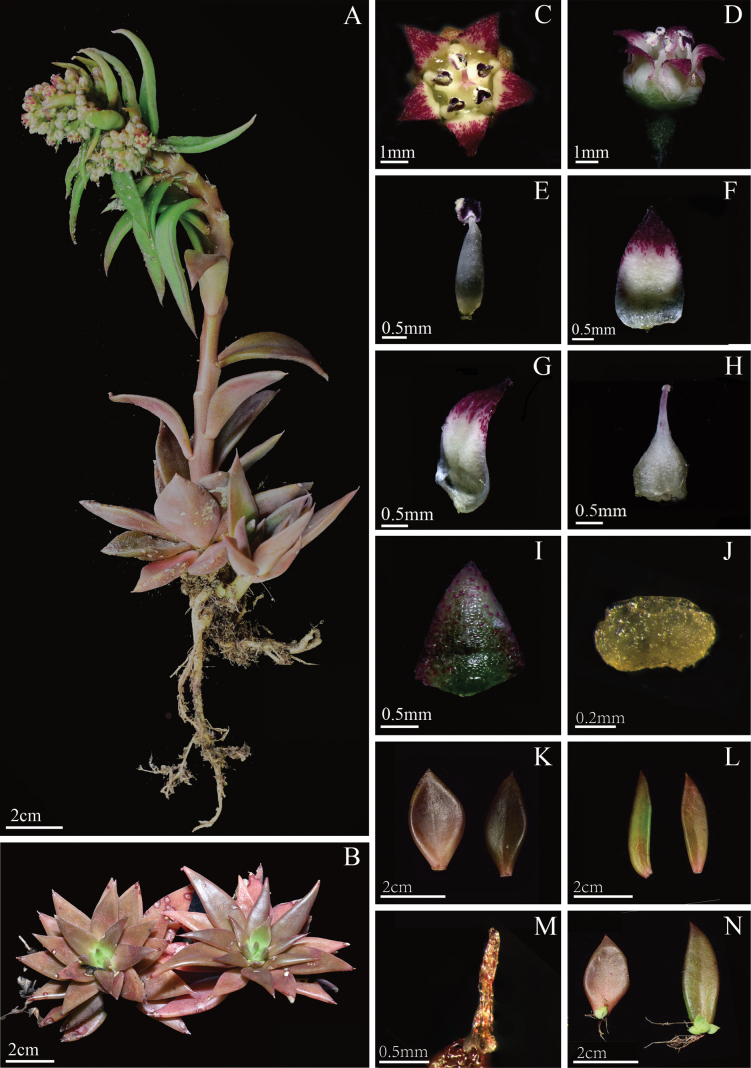
*Sinocrassulaobliquifolia***A, B** habit **C, D** flowers **E** stamen **F, G** petals **H** carpel **I** sepal **J** nectar scale **K** basal leaves **L** stem leaves **M** gland of basal leaf **N** leaves that abscise and bud during the flowering period, left: basal leaf, right: stem leaf.

The new species exhibits some typical characteristics of *Sinocrassula* including compact rosette, stem leaves alternate and lanceolate, bracts resembling distal stem leaves but smaller, flowers 5-merous, broadly triangular sepals, and ovoid carpels (Fu and Ohba 2013). A comparison of morphological characters among the new species and its morphologically similar species is shown in Table 2. The new species can easily distinguished from other species by its unique combination of asymmetrical leaves, rectangler nectar scales, and triangular petals which are white at the base and gradually transition to purple toward the tips (Fig. 2).

## ﻿Taxonomic treatment

### ﻿ Sinocrassula obliquifolia

Taxon classificationPlantaeSaxifragalesCrassulaceae

Jing Zhao, J.Guan Wang & X.M.Zhou sp. nov.

8D4FAA01-BA72-595C-BF6A-80F7BBF3D03D

urn:lsid:ipni.org:names:77360183-1

Figs 2 , 3

#### Type.

China • Sichuan: Hanyuan County, Yaan City, elev. ca. 837 m, 29.333262°N, 102.571721°E, on the granite crevices, 6 July 2023, *Jing Zhao et al. YUS9064* (holotype YUKU!; isotype YUKU!).

**Figure 3.**
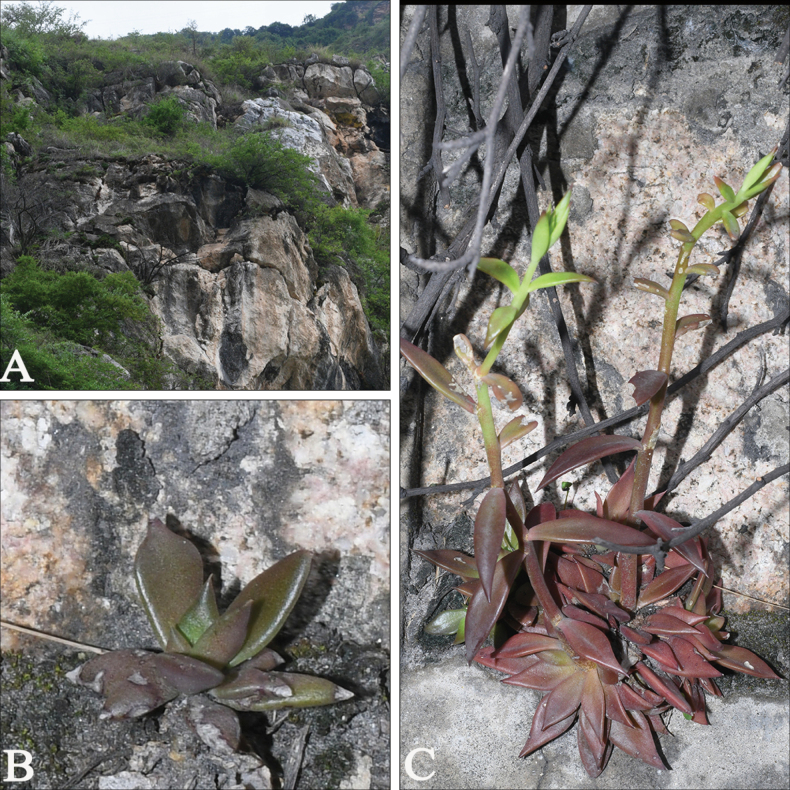
*Sinocrassulaobliquifolia***A** habitat **B, C** habit.

#### Diagnosis.

*Sinocrassulaobliquifolia* is similar to *S.indica* in having rosette, ovoid carpels, and broadly triangular sepals. However, this new species has shorter flowering stem (10–18 cm) (vs. 5–60 cm in *S.indica*), lanceolate stem leaves (vs. oblanceolate to ovate-orbicular stem leaves in *S.indica*), triangular petals (vs. lanceolate to ovate petals in *S.indica*), petals with a white base adorned with dense purple-red stripes and spots on the surfaces upward (vs. red, reddish, yellow, or greenish-yellow in *S.indica*) and rectangle nectar scales (vs. quadrate in *S.indica*). *S.obliquifolia* is also similar to *S.diversifolia* in having corymbiform inflorescences, petals, and stamens with purple spots. However, *S.obliquifolia* has a well-defined rosette (vs. less defined rosette in *S.diversifolia*), monomorphic stem leaves (vs. dimorphic stem leaves in *S.diversifolia*), triangular petals with dense purple-red stripes and spots on the surfaces upward (vs. lanceolate petals that are yellow with purple spots in *S.diversifolia*) and rectangle nectar scales (vs. broadly quadrate in *S.diversifolia*).

#### Description.

Perennial herbs, terrestrial or lithophytic, 5.0–20.0 cm tall, rosette 5.0–8.0 × 6.0–8.0 cm. Roots fibrous. Basal leaves rosette, spirally arranged, asymmetrically ovoid to lanceolate, apex with glands, 2.0–3.0 × 1.0–2.0 cm. Flowering stems terminal, 10.0–18.0 cm, glabrous with purple spots. Stem leaves alternate, lanceolate, many parts with purple spots. Bract resembling distal stem leaves but smaller, lanceolate, 3.0–3.5 × 0.5–1.0 cm. Inflorescences corymbiform, ca. 2.0–3.0 cm in diameter. Flowers small, ca. 3–6 mm in diameter. Sepals broadly triangular, purple with red spots, 1.5–2.0 × 0.5–1.2 mm. Petals triangular, base white, with dense purple-red stripes and spots on the surfaces upward, 2.0–4.0 × 1.0–2.0 mm. Stamens ca. 2–3 mm, white, anthers oblong-cordate, ca. 0.5 mm, pollen yellow. Nectar scales broadly rectangular, ca. 0.3–0.5 × 0.2–0.3 mm. Carpels 5, ovoid, clockwise rotation, 1.0–2.0 × 0.5–1 mm, styles 1.0–1.5 mm. Flowering June–October which coincides with the rainy season and the shedding of the leaves and the emergence of new buds.

#### Distribution and habitat.

*Sinocrassulaobliquifolia* is currently known in central Sichuan Province, China. Two populations were found in granite crevices, as well as on dry stony or gravelly slopes at elevations ranging from 837 to 1140 m.

#### Additional specimens examined (paratypes).

China • Sichuan: Shimian County, Yaan City, elev. ca. 1140 m, 29.258594°N, 102.371583°E, on the granite crevices, 12 May 2023, *Chuan-Jie Huang et al. YUS9366* (YUKU!).

#### Etymology.

The epithet *obliquifolia* refers to the asymmetrical leaves of the basal leaves, a unique characteristic for this species within *Sinocrassula*. Its Chinese name is suggested as ‘斜叶石莲(xie ye shi lian)’.

## Supplementary Material

XML Treatment for Sinocrassula obliquifolia

## ﻿Additional information

### Author ORCIDs

Rong-Juan Li https://orcid.org/0009-0008-2667-398X

Jing Zhao https://orcid.org/0000-0001-7871-2209

Shao-Li Fang https://orcid.org/0000-0001-5942-0366

Chuan-Jie Huang https://orcid.org/0009-0000-3507-2766

Miao Luo https://orcid.org/0009-0006-9317-9151

Zhao-Rong He https://orcid.org/0000-0001-6492-3689

Xin-Mao Zhou https://orcid.org/0000-0003-3555-7784

Jia-Guan Wang https://orcid.org/0000-0003-0264-9180

### Data availability

All of the data that support the findings of this study are available in the main text.
